# Postoperative Tourniquet Pain in Patients Undergoing Foot and Ankle Surgery

**DOI:** 10.7759/cureus.3678

**Published:** 2018-12-04

**Authors:** Promil Kukreja, Eva Lehtonen, Martim C Pinto, Harshadkumar A Patel, Haley M McKissack, Ashish Shah

**Affiliations:** 1 Anesthesiology, University of Alabama School of Medicine, Birmingham, USA; 2 Orthopaedics, University of Miami Miller School of Medicine, Miami, USA; 3 Orthopaedics, University of Alabama at Birmingham, Birmingham, USA; 4 Orthopaedics, University of Alabama School of Medicine, Birmingham, USA

**Keywords:** tourniquet pressure, tourniquet time, pain, foot and ankle surgery

## Abstract

Background

Tourniquets are commonly used to reduce bleeding intraoperatively during orthopedic surgery. There are variable guidelines for ideal tourniquet pressure and duration; the practice of fixed, high tourniquet pressures remains common. The purpose of this study was to assess the correlation between excessive tourniquet pressure and duration and the incidence of tourniquet pain in foot and ankle surgery patients.

Methods

A retrospective cohort study was conducted on 128 patients who underwent foot and ankle surgery with tourniquet use. Baseline systolic blood pressure (SBP), tourniquet pressure and duration, intraoperative opioid consumption, post-anesthesia care unit (PACU) pain scores, PACU opioid consumption, and PACU length of stay (LOS) were collected. Linear regression analysis was used to test for the statistical correlation between the tourniquet pressure and duration and postoperative pain scores, narcotic use, and PACU LOS.

Results

A tourniquet pressure of 280 mmHg was used in 90% of the cases (*N *= 128). Only 2.5% of the patients had tourniquet pressures 100-150 mmHg above SBP. The mean tourniquet time was 107.5 minutes ± 39.8. Linear regression showed a significant positive correlation between tourniquet time and morphine equivalents used in the perioperative period (*r *= 0.410; *p *< 0.001) and the length of PACU stay (*r *= 0.250; *p *= 0.012).

Conclusion

Prolonged tourniquet times at high pressures, not based on limb occlusion pressure LOP, lead to increased pain and opioid use and prolonged PACU LOS. Basing tourniquet pressures on LOPs could likely improve the safety margin of the tourniquets; however, randomized clinical trials are needed.

## Introduction

The tourniquet is commonly used in orthopedic surgeries on the upper and lower extremities to reduce blood loss, improve visualization, and expedite the surgical procedure. However, tourniquets have been associated with multiple local and systemic complications causing significant morbidity to patients. Tourniquets can cause pain, paralysis, damage to local skin, vasculature or neuromuscular structures, thrombosis and pulmonary embolism, compartment syndrome, reperfusion syndrome, and tourniquet pain syndrome [1]. Rarely, patients can even experience permanent damage or loss of limb function secondary to tourniquet use [1].

Patients who receive sufficient surgical block with regional anesthesia can still experience tourniquet pain postoperatively [1]. The etiology of tourniquet-related pain is still being investigated, but it is likely mediated by nerve ischemia and compression of unmyelinated C-fibers, which play an important role in pain pathways and are responsible for dull pain [2]. The smaller unmyelinated C-fibers are more resistant to local anesthetic-induced conduction block as compared to the larger myelinated A-fibers [3]. After intrathecal administration of an adequate dose of local anesthetic, conduction in both A- and C-fibers is blocked [4]. As the concentration of local anesthetic in the cerebrospinal fluid decreases, the C-fibers regain conduction of impulses before the A-fibers, resulting in a dull tourniquet pain in the presence of an anesthetic. Tourniquet pain is reported to develop in up to 66% of patients, 30 to 60 minutes after cuff inflation in patients receiving regional anesthesia to the arm or leg [5]. In a study involving awake patients, increased blood pressure (BP) correlated with the development of pain caused by the tourniquet [6]. After surgery, a patient may complain of a dull, achy pain at the site of the tourniquet had been previously placed [7]. In the majority of patients, this pain resolves but a patient could have delayed muscle rehabilitation secondary to irreversible nerve ischemia [8]. A 2006 Norwegian study placed nerve complication incidence as high as 1 in 4,232 operations [9].

Unfortunately, there is a lack of consensus in the surgical community regarding safe and effective tourniquet pressure and duration of use, and practice remains highly variable. The Association of Surgical technologists recommends tourniquet pressures of 100 mmHg above systolic blood pressure (SBP) for the lower extremity, while Kam et al. suggest that a range of 100-150 mmHg above the SBP is acceptable and Wakai et al. expands this range to 90-150 mmHg above SBP for the lower extremity or 50-75 mmHg above the limb occlusion pressure (LOP) [2,6,10]. Noordin et al. recommend using the LOP, which is calculated using Graham’s formula for minimal arterial occlusion pressure (AOP) “AOP = [(systolic pressure – diastolic pressure) (limb circumference)/3(cuff width)] + diastolic pressure” [11]. Despite these recommendations, many surgeons prefer to use fixed tourniquet pressures, often 250 mmHg or higher [6]. A better understanding of tourniquet use and safety is paramount to create standards of practice, which reduce the healthcare burden of complications secondary to tourniquet use. 

This retrospective study aims to add to the literature by assessing for the correlation between excessive tourniquet pressure and duration and the increased incidence of tourniquet pain in foot and ankle patients. With increased awareness of the potential complications that tourniquets play in the role of postoperative pain, new parameters regarding tourniquet use intra-operatively can lead to decreased incidence of tourniquet side effects and potentially lead to a change in our practice regarding tourniquet use.

## Materials and methods

This study was approved by the University of Alabama at Birmingham’s (UAB) Institutional Review Board. Patients who met the following criteria were included in this study: 18 years of age or older, underwent foot and ankle surgery, received a preoperative regional nerve block for postoperative analgesia, and received intraoperative use of a tourniquet. We excluded patients who had a history of daily opioid use of more than 30 mg oral morphine equivalents (OME) for more than 30 days, with the preexisting preoperative pain score greater than five out of 10 on the visual analog scale (VAS), underwent foot and ankle surgery without a regional nerve block, were deemed regional block failures by the regional anesthesia provider, did not have the peripheral nerve block type recorded, or underwent foot and ankle surgery without tourniquet use.

Chart review and data extraction

Data were extracted by a single, independent author. The variables of interest for this study were patient’s baseline SBP, tourniquet pressure and duration, tourniquet deflation time, tourniquet reinflation pressure and duration, start time of BP and heart rate changes, intraoperative opioid consumption before BP and heart rate changes, intraoperative opioid consumption after BP and heart rate changes, BP and heart rate changes after deflation, post-anesthesia care unit (PACU) pain scores, PACU opioid consumption, and PACU length of stay (LOS). Opioid use was converted to OME for a standardized comparison.

Statistics

Statistical analysis was performed using SPSS version 25 (IBM, Chicago, IL). Demographic and clinical variables with continuous measures were expressed as means and standard deviations; categorical variables were expressed as proportions. Continuous data were analyzed using the Kolmogorov-Smirnov test (non-normally distributed) and the one-way analysis of variance (ANOVA) and Student’s *t*-test (normally distributed). For data that are not normally distributed, the Kruskal-Wallis and Mann-Whitney tests were used for comparisons. Chi-square and Fisher’s exact tests were used to analyze categorical data. Pearson correlation test and linear regression statistics were used to test the relationship between pain scores and tourniquet use parameters, while controlling for relevant clinical and demographic variables. A *p*-value of <0.05 was considered statistically significant.

## Results

Between August 2015 and December 2015, 128 patients (49 males) met the study inclusion criteria. The patient characteristics of these 128 patients are shown in Table 1. The most commonly used peripheral nerve block types were popliteal (75 patients) and sciatica (37 patients). The most common types of surgery were ankle procedures (30 patients), hindfoot procedures (30 patients), and combined ankle and hindfoot (26 patients) procedures.

**Table 1:** Patient characteristics

Age* (years)	47.6 +/- 14.8 (16-9)
Sex	
Male	49 (38%)
Female	79 (62%)
American Society of Anesthesiologists (ASA) Classification	
1	9 (7.0%)
2	61 (47.7%)
3	57 (44.5%)
4	1 (0.8%)
Side of Surgery	
Left	50 (40%)
Right	75 (60%)
Surgery Site	
Ankle (A)	30 (23.4%)
Hindfoot (H)	30 (23.4%)
Midfoot (M)	7 (5.5%)
Forefoot (F)	11 (8.6%)
A + H	26 (20.3%)
F + M	13 (10.3%)
A + M	2 (1.6%)
H + M	1 (0.8%)
A + H + M	6 (4.7%)
Other	2 (1.6%)
*Values given as Mean +/- standard deviation and (range). #Values given as number of patients (percent of total)	

Outcomes

The mean opioid use before hemodynamic changes was 34 mg OME (range, 7.5-120 mg) and after hemodynamic changes 12.5 mg OME (range, 0-85 mg).

As seen in Table 2, the mean tourniquet time was 107.5 minutes ±39.8; (range, 16-221). Patients with long tourniquet times (≥90 minutes) had significantly greater mean intraoperative opioid use (18.9 mg ± 22.1; range, 0-85) as compared to patients with short tourniquet times (<90 minutes; 4.1 mg ± 11.8 [range, 0-52.5 mg]; *p* < 0.001).

**Table 2:** Tourniquet time Long tourniquet times (≥90 minutes) were associated with higher amounts of intraoperative pain medications administered (mg of OME) than short tourniquet times (<90 minutes). *The amount of pain medications are expressed in milligrams of OME. The amounts depicted in this table are amounts given after the hemodynamic change that was suspected to be due to tourniquet pain. OME: oral morphine equivalents

Tourniquet Time	N	Mean (mg)*	SD (mg)	*P*-value
Long	82	19.0	22	<0.001
Short	48	4.8	11.6	<0.001

**Figure 1:**
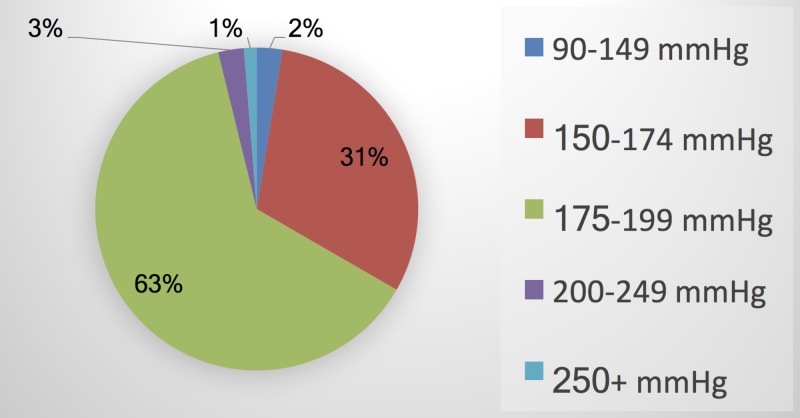
Pneumatic tourniquet pressures greater than systolic blood pressure used during foot and ankle surgery at UAB Highlands Hospital UAB: University of Alabama at Birmingham

The mean tourniquet pressure for all patients was 280.1 mmHg ± 6.5 (range, 250-300 mmHg). Tourniquet pressure was 280 mmHg in 90% of patients in the "long" tourniquet group (74 of 82 patients). Among the 81 patients for whom both SBP and tourniquet pressure were available, the mean tourniquet pressure above SBP was 176.6 mmHg ± 15.3 (range,122-210). Only 2% of patients had tourniquet pressures within 90 to 149 mmHg above SBP (Figure 1).

The mean PACU LOS was 68.3 minutes ± 28.3 (range, 20-205 minutes). PACU discharge pain scores were available for 33 patients; the mean pain score at discharge was 2 ± 2.4 (range, 0-6 out of 10).

**Figure 2:**
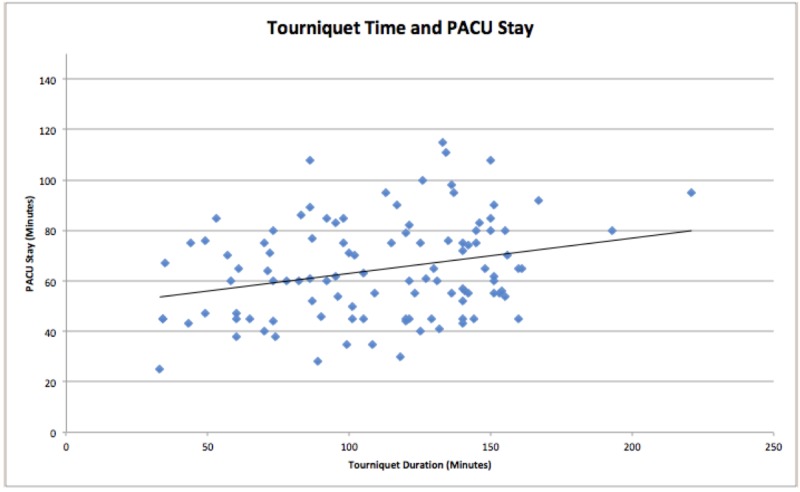
Positive linear correlation between tourniquet time and PACU length of stay PACU: post-anesthesia care unit

Pearson correlation test showed a significant positive correlation between the tourniquet time and the OMEs used in the perioperative period (*N *= 117; *r* = 0.410; *p* < 0.001) and PACU LOS (*N *= 100; *r *= 0.250; *p* = 0.012) as shown in Figure 2. No statistically significant correlation was observed between the tourniquet time and the PACU discharge pain score (*N *= 30; *r *= 0.026; *p *= 0.891).

Tourniquet pressure was not significantly correlated with OMEs used in the perioperative period (*N* = 82; *r* = 0.100; *p* = 0.369), PACU LOS (*N* = 68; *r* = -0.145; *p* = 0.236), or PACU discharge pain scores (*N* = 27; *r* = 0.094; *p* = 0.640).

There was no significant correlation between the difference between the tourniquet pressure and the patient’s SBP and the morphine equivalents used in the perioperative period (*N* = 81; *r* = 0.213; *p* = 0.056), PACU LOS (*N* = 68; *r* = 0.172; *p* = 0.160), or PACU discharge pain scores (*N *= 26; *r* = -0.365; *p* = 0.066). 

This work was presented at the 2018 American Orthopaedic Foot and Ankle Society meeting (Abstract: Ashish Shah, Eva Lehtonen, Samuel Huntley, Harshadkumar Patel, John Johnson, Zachariah Pinter, Sameer Naranje, Sung Lee, Promil Kukreja, Ilya Gutman, BS. Postoperative Tourniquet Pain in Patients Undergoing Foot and Ankle Surgery. American Orthopaedic Foot & Ankle Society; September 18, 2018).

## Discussion

Tourniquets are used in orthopedic surgeries to reduce bleeding, but postoperative pain is among the possible complications. Various guidelines exist regarding ideal tourniquet pressure and duration, but the practice of fixed, high tourniquet pressures remains common. The purpose of this study was to assess the correlation between excessive tourniquet pressure and duration and the increased incidence of tourniquet pain in foot and ankle surgery patients.

Tourniquet time was found to positively correlate with greater opioid use in the perioperative period and longer duration of stay in the PACU. Long tourniquet times (≥90 minutes) were also associated with significantly greater intraoperative opioid use than short tourniquet times (<90 minutes). These findings are consistent with previous studies in the orthopedic literature. A retrospective review (*N *= 603) by Kruse et al. comparing postoperative opioid use in first 24 hours in patients undergoing total knee replacement with and without the use of intraoperative tourniquet showed an increased tourniquet time was associated with increased opioid use, with every additional 10 minutes of tourniquet time leading to an additional 0.43 mg of opioid consumption [12]. This phenomenon may be mediated by ischemia and reperfusion injury following tourniquet use leading to higher postoperative pain and subsequently higher opioid use during the postoperative period [13-14]. Interestingly, however, our study did not demonstrate an association between tourniquet duration and discharge pain scores. The low response rate for discharge pain scores may be responsible for losing this association, which has been established in previous studies. However, postoperative pain is dependent on various factors, besides tourniquet pressure and duration, such as age, gender, and preexisting pain that may impact the severity of postoperative pain.

Our study also showed that a longer tourniquet time was associated with a longer PACU stay. The literature available on foot and ankle is less, but the association of tourniquet time and the length of hospital stay has been reported in several studies of total knee replacements. Olivecrona et al. reported that longer tourniquet time (>100 minutes) leads to more postoperative complications and longer hospital stay [15]. By contrast, Raut et al. reported that tourniquet time was not a significant factor affecting the prolonged length of hospital stay following total knee replacement [16]. Although this study found a correlation between tourniquet time and PACU LOS, this outcome depends on many factors, including the availability of beds, nurses, and case volume and complexity, among other variables. Anecdotally, opioid administration extends the PACU LOS at our institution by about 30 minutes, which may explain this correlation.

No strict consensus exists on ideal tourniquet time. The existing literature recommends 1 to 3 hours as a safe limit for tourniquet time, and if the anticipated surgery time is >2.5 h, the tourniquet should be deflated for 10-15 minutes before re-inflating again [7]. Other studies recommend that tourniquet inflation times for pediatric patients should be kept below 75 minutes [17-18].

This study found poor compliance with the tourniquet use guidelines at our institution. Tourniquet pressure was 280 mmHg in 90% of patients, while only 2.5% of the patients had tourniquet pressures between 100 and 150 mmHg above SBP. These findings were in line with a survey by Delougry et al. who reported that the majority of orthopedic surgeons were not aware of the used tourniquet pressure and preferred to use fixed pressures for upper extremity (250 mmHg) and lower extremity (300 mmHg) procedures, without considering the patient’s baseline blood pressure [5]. As younger patients tend to have lower systolic blood pressures, the use of fixed tourniquet pressure leads to a larger difference between the LOP and tourniquet pressure in younger patients. This ultimately results in higher compression pressure and tourniquet-related complications [19].

The clinical implications of widespread improper tourniquet use should not be underestimated. Increased perioperative opioid consumption has been shown to correlate with hyperalgesia and an increased opioid tolerance [20-21]. Tourniquet pressure based on LOP or SBP is therefore recommended to minimize the risk of tourniquet-related complications [22-23]. Efforts aimed to increase surgeon awareness of the tourniquet use guidelines and the consequences of using fixed, high-pressure tourniquets are needed and may lead to improved patient outcomes and reduced healthcare costs.

Although this study was unable to find a statistically significant correlation between the difference of tourniquet pressure and SBP and opioid use in the perioperative period, it seems plausible that such a relationship could exist. Thus, this lack of a finding may represent a type II error caused by the small number of patients (*N *= 82) in whom both tourniquet pressure and SBP were recorded. Larger studies are needed to establish the presence or absence of relevant correlations between this pressure difference and opioid use, LOS, and complication rates. 

The primary limitations of this study were those inherent to all retrospective studies. In addition, this study was limited by a lack of spread of tourniquet pressures, which likely limited its ability to find the correlations between the tourniquet pressure and postoperative pain, opioid use, or PACU LOS. Opioid use may have confounded the associations observed between the tourniquet time and PACU LOS, due to the delay caused by the administration of opioids. Finally, the study was limited by missing outcomes data that may have reduced the statistical power and significant correlations.

## Conclusions

The majority of cases of foot and ankle surgery at our institution did not adhere to the current tourniquet use guidelines, which recommend a tourniquet pressure between 100 and 150 mmHg above the patient's SBP. Prolonged tourniquet times at high pressures not based on LOP, as observed in our study, lead to increased pain and opioid use postoperatively and prolonged time in the PACU. Basing tourniquet pressures on LOPs could likely improve the safety margin of tourniquet use in foot and ankle surgery; however, future investigations in the form of prospective randomized studies are warranted.

## References

[1] Van der Spuy L (2012). Complications of the arterial tourniquet. South Afr J Anaesth.

[2] Kam PC, Kavanagh R, Yoong FF (2001). The arterial tourniquet: pathophysiological consequences and anaesthetic implications. Anaesthesia.

[3] Concepcion MA, Lambert DH, Welch KA, Covino BG (1988). Tourniquet pain during spinal anesthesia: a comparison of plain solutions of tetracaine and bupivacaine. Anesth Analg.

[4] Gissen AJ, Covino BG, Gregus J (1980). Differential sensitivities of mammalian nerve fibers to local anesthetic agents. Anesthesiology.

[5] Deloughry JL, Griffiths R (2009). Arterial tourniquets. Contin Educ Anaesth Crit Care Pain.

[6] Fitzgibbons PG, Digiovanni C, Hares S, Akelman E (2012). Safe tourniquet use: a review of the evidence. J Am Acad Orthop Surg.

[7] Hagenouw RR, Bridenbaugh PO, van Egmond J, Stuebing R (1986). Tourniquet pain: a volunteer study. Anesth Analg.

[8] Nitz AJ, Matulionis DH (1982). Ultrastructural changes in rat peripheral nerve following pneumatic tourniquet compression. J Neurosurg.

[9] Odinsson A, Finsen V (2006). Tourniquet use and its complications in Norway. J Bone Joint Surg.

[10] Wakai A, Winter DC, Street JT, Redmond PH (2001). Pneumatic tourniquets in extremity surgery. J Am Acad Orthop.

[11] Noordin S, McEwen JA, Kragh JF, Eisen A, Masri BA (2009). Surgical tourniquets in orthopaedics. J Bone Joint Surg Am.

[12] Kruse H, Christensen KP, Moller AM, Gogenur I (2015). Tourniquet use during ankle surgery leads to increased postoperative opioid use. J Clin Anesth.

[13] Omeroglu H, Gunel U, Bicimoglu A, Tabak AY, Ucaner A, Guney O (1997). The relationship between the use of tourniquet and the intensity of postoperative pain in surgically treated malleolar fractures. Foot Ankle Int.

[14] Konrad G, Markmiller M, Lenich A, Mayr E, Ruter A (2005). Tourniquets may increase postoperative swelling and pain after internal fixation of ankle fractures. Clin Orthop Relat Res.

[15] Olivecrona C, Lapidus LJ, Benson L, Blomfeldt R (2013). Tourniquet time affects postoperative complications after knee arthroplasty. Int Orthop.

[16] Raut S, Mertes SC, Muniz-Terrera G, Khanduja V (2012). Factors associated with prolonged length of stay following a total knee replacement in patients aged over 75. Int Orthop.

[17] Sharma JP, Salhotra R (2012). Tourniquets in orthopedic surgery. Indian J Orthop.

[18] Kumar K, Railton C, Tawfic Q (2016). Tourniquet application during anesthesia: “What we need to know?”. J Anaesthesiol Clin Pharmacol.

[19] Horlocker TT, Hebl JR, Gali B (2006). Anesthetic, patient, and surgical risk factors for neurologic complications after prolonged total tourniquet time during total knee arthroplasty. Anesth Analg.

[20] Chu LF, Clark DJ, Angst MS (2006). Opioid tolerance and hyperalgesia in chronic pain patients after one month of oral morphine therapy: a preliminary prospective study. J Pain.

[21] Mercadante S, Ferrera P, Villari P, Arcuri E (2003). Hyperalgesia: an emerging iatrogenic syndrome. J Pain Symptom Manag.

[22] Guay J (2009). Adverse events associated with intravenous regional anesthesia (Bier block): a systematic review of complications. J Clin Anesth.

[23] Hicks RW, Denholm B (2013). Implementing AORN recommended practices for care of patients undergoing pneumatic tourniquet-assisted procedures. AORN J.

